# Supplementary material for: The Stability of Social and Behavioral Rhythms and Unexpected Low Rate of Relevant Depressive Symptoms in Old Adults during the COVID-19 Pandemic
Source: J Clin Med. 2024 Mar 29;13(7):2005. doi: 10.3390/jcm13072005 (PMC11012795; doi:10.3390/jcm13072005)
Supplement: Supplementary file 1 [file jcm-13-02005-s001.zip › BSRS_italian.pdf]

SCALA BREVE SUI RITMI SOCIALI

Adattamento italiano da: Jürgen Margraf, Kristen Lavallee, XiaoChi Zhang, Silvia Schneider, PLoS ONE, 2016; 11(3): e0150312. doi:10.1371/journal.pone.0150312

Le affermazioni che seguono sono relative ai ritmi e alle attività quotidiane nella tua vita. Ti chiediamo di indicare il livello di regolarità con cui porti avanti ciascuna delle attività.

	Molto regolare	Abbastanza regolare	Un po' regolare	Un po' irregolare	Abbastanza irregolare	Molto irregolare
1. Andare a dormire (dal Lunedì al Venerdì)						
2. Andare a dormire (nel fine settimana)						
3. Svegliarsi ed alzarsi dal letto (dal Lunedì al Venerdì)						
4. Svegliarsi ed alzarsi dal letto (nel fine settimana)						
5. Incontrare altre persone a scuola o al lavoro (dal Lunedì al Venerdì)						
6. Incontrare altre persone a scuola o al lavoro (nel fine settimana)						
7. Incontrare altre persone nel tempo libero (dal Lunedì al Venerdì)						
8. Incontrare altre persone nel tempo libero (nel fine settimana)						
9. Mangiare i pasti principali (dal Lunedì al Venerdì)						
10. Mangiare i pasti principali (nel fine settimana)						
