# Supplementary material for: The Stability of Social and Behavioral Rhythms and Unexpected Low Rate of Relevant Depressive Symptoms in Old Adults during the COVID-19 Pandemic
Source: J Clin Med. 2024 Mar 29;13(7):2005. doi: 10.3390/jcm13072005 (PMC11012795; doi:10.3390/jcm13072005)
Supplement: Supplementary file 1 [file jcm-13-02005-s001.zip › PHQ-9-Italian.pdf]

PHQ9P

QUESTIONARIO SULLA SALUTE DEL/DELLA PAZIENTE-9
(Italian version of the PHQ-9)

72883

THIS SECTION FOR USE BY STUDY PERSONNEL ONLY.

Were data collected? **No** ☐ *(provide reason in comments)*

If **Yes**, data collected on visit date ☐ **or** specify date: ___________________
DD-Mon-YYYY

Comments:

Only the patient (subject) should enter information onto this questionnaire.

**Nelle <u>ultime 2 settimane</u>, con quale frequenza le ha dato fastidio ciascuno dei seguenti problemi?**	**Mai**	**Alcuni giorni**	**Per più della metà del tempo**	**Quasi ogni giorno**
1. Scarso interesse o piacere nel fare le cose	0	1	2	3
2. Sentirsi giù, triste o disperato/a	0	1	2	3
3. Problemi ad addormentarsi o a dormire tutta la notte senza svegliarsi, o a dormire troppo	0	1	2	3
4. Sentirsi stanco/a o avere poca energia	0	1	2	3
5. Scarso appetito o mangiare troppo	0	1	2	3
6. Avere una scarsa opinione di sé, o sentirsi un fallimento o aver deluso se stesso/a o i propri familiari	0	1	2	3
7. Difficoltà a concentrarsi su qualcosa, per esempio leggere il giornale o guardare la televisione	0	1	2	3
8. Muoversi o parlare così lentamente da poter essere notato/a da altre persone. O, al contrario, essere così irrequieto/a da muoversi molto più del solito	0	1	2	3
9. Pensare che sarebbe meglio morire o farsi del male in un modo o nell'altro	0	1	2	3

SCORING FOR USE BY STUDY PERSONNEL ONLY

0 + ______ + ______ + ______

=Total Score: ______

Se ha fatto una crocetta su uno <u>qualsiasi</u> di questi problemi, quanto questi problemi le hanno reso <u>difficile</u> fare il suo lavoro, occuparsi delle sue cose a casa o avere buoni rapporti con gli altri?

**Per niente difficile**	**Abbastanza difficile**	**Molto difficile**	**Estremamente difficile**
☐	☐	☐	☐

Copyright © 2005 Pfizer Inc. Tutti i diritti riservati. Riprodotto con autorizzazione. EPI0905.PHQ9P

Confermo l'esattezza di queste informazioni.	Iniziali del/della paziente o del soggetto:	Data:
